# Designing Messaging to Engage Patients in an Online Suicide Prevention Intervention: Survey Results From Patients With Current Suicidal Ideation

**DOI:** 10.2196/jmir.3173

**Published:** 2014-02-07

**Authors:** Ursula Whiteside, Anita Lungu, Julie Richards, Gregory E Simon, Sarah Clingan, Jaeden Siler, Lorilei Snyder, Evette Ludman

**Affiliations:** ^1^Group Health Research InstituteSeattle, WAUnited States

**Keywords:** human centered design, user centered design, health 2.0, suicide

## Abstract

**Background:**

Computerized, Internet-delivered interventions can be efficacious; however, uptake and maintaining sustained client engagement are still big challenges. We see the development of effective engagement strategies as the next frontier in online health interventions, an area where much creative research has begun. We also argue that for engagement strategies to accomplish their purpose with novel targeted populations, they need to be tailored to such populations (ie, content is designed with the target population in mind). User-centered design frameworks provide a theoretical foundation for increasing user engagement and uptake by including users in development. However, deciding how to implement this approach to enage users in mental health intervention development is challenging.

**Objective:**

The aim of this study was to get user input and feedback on acceptability of messaging content intended to engage suicidal individuals.

**Methods:**

In March 2013, clinic intake staff distributed flyers announcing the study, “Your Feedback Counts” to potential participants (individuals waiting to be seen for a mental health appointment) together with the Patient Health Questionnaire. The flyer explained that a score of two or three (“more than half the days” or “nearly every day” respectively) on the suicide ideation question made them eligible to provide feedback on components of a suicide prevention intervention under development. The patient could access an anonymous online survey by following a link. After providing consent online, participants completed the anonymous survey.

**Results:**

Thirty-four individuals provided data on past demographic information. Participants reported that they would be most drawn to an intervention where they knew that they were cared about, that was personalized, that others like them had found it helpful, and that included examples with real people. Participants preferred email invitations with subject lines expressing concern and availability of extra resources. Participants also provided feedback about a media prototype including a brand design and advertisement video for introducing the intervention.

**Conclusions:**

This paper provides one model (including development of an engagement survey, audience for an engagement survey, methods for presenting results of an engagement survey) for including target users in the development of uptake strategies for online mental health interventions.

## Introduction

Internet-based interventions have the potential to increase the accessibility and efficacy of mental health treatment [1-4]. They are effective in producing change; several meta-analyses have found no significant differences in results when compared to face-to-face interventions [1,5,6]. Most of the current, long-established, research-based mental health treatment options serve a population that is already either seeking treatment and receiving care, or is in crisis and getting services from the emergency department [7]. But the Internet helps us broaden the populations being served and reach individuals who might otherwise be missed in more traditional delivery modalities [8]. The Internet can reach those not seeking traditional care due to logistic difficulties in accessing treatment or stigma around mental illness and has potential for large scale dissemination [9]. However, unsatisfactory uptake and follow-through is a significant barrier to reaching the full potential of online interventions [10-13]. Thus, identifying effective strategies to engage the target population is of critical importance for Internet-based mental health interventions to reach their optimum potential.

The involvement of consumer collaborators in mental health research has the potential to transform care and increase patient engagement [14]. Yet, the peripheral role of the end-user in the development of online mental health interventions has been proposed as a major barrier to utilizing such a service or program [15]. Health 2.0 (and Medicine 2.0, ie, the application of Web 2.0 technologies to health care) and human-centered design (a design process extensively relying on the intended user at all levels of the design process) are useful frameworks for enabling users to have an active role in generating and manipulating Web content and participating in health promotion [16,17]. These frameworks provide a theoretical foundation for increasing user engagement and uptake, but deciding how to implement this approach to increase engagement in mental health interventions is challenging.

Suicide is a devastating consequence of mental illness and major mental health concern in the United States, ranking as the 10^th^ leading cause of death and generating high-cost emergency room visits and hospitalizations [18]. Research has led to the production of mental health interventions efficacious in treating suicidality [19-21], including a Dutch online program designed specifically to decrease frequency and intensity of suicidal ideation [22,23]. However, results from the Dutch study indicate that over half the eligible patients did not return the study consent form [22], thus reinforcing the importance of user engagement.

The aim of this project was to take the first step in attempting to increase user uptake and engagement in an online suicide prevention study by soliciting feedback from patients eligible for the intervention program. Specifically, we surveyed patients with current suicidal ideation about acceptable subject lines, intervention descriptions, project names, and introductory videos in order to gain a better understanding of how to reach the target audience.

## Methods

### Participant Recruitment

Individuals seen for mental health treatment at Group Health, a large health care organization based in Seattle, Washington, are asked to complete the Patient Health Questionnaire (PHQ-9) before every visit as part of routine care. The PHQ-9 is a 9-item, self-report questionnaire used for screening, monitoring, and measuring the severity of depression over the previous 2 weeks [24]. The PHQ-9 uses a 4-point scale (“not at all”, “several days”, “more than half the days”, “nearly every day”), and the last question asks about presence and duration of suicide ideation. An answer of “more than half the days” or “nearly every day” for suicide ideation has been found to predict subsequent suicide attempts or suicide death [25].

In March 2013, clinic intake staff distributed flyers announcing the study, “Your Feedback Counts”, to potential participants (individuals waiting to be seen for mental health appointments) together with the PHQ-9. The flyer explained that a score of two or three (“more than half the days” or “nearly every day” respectively) on the suicide ideation question made them eligible to provide feedback on components of a suicide prevention intervention under development. The patient could access an anonymous online survey by following a link. After providing consent online, participants completed the anonymous survey.

As a last step, participants could link to a separate survey and provide their contact information to receive a US $10 incentive. No link between survey data and contact information was collected. All study procedures were approved by the Group Health Institutional Review Board.

### Survey

The survey included questions about demographics and medical treatment for a suicide attempt or self-injury, as well as acceptability of proposed intervention messaging (see Multimedia Appendices 1 and 2). A patient consultant group of 5 individuals with a history of suicidal ideation and suicide attempts helped to generate the messaging options, as well as a video describing the intervention. There were 26 options for message subject lines introducing the intervention, 20 options for intervention characteristics, 9 intervention brand names, and an option for study participants to make additional suggestions. The survey was programmed using DatStat Ilume and accessible by computer or other portable Internet-connected device (smartphone, iPad, etc) [26]. All questions related to the intervention invitation and description were asked using a 5-point Likert scale (0=strongly disagree; 4=strongly agree). In evaluating the results, we calculated the difference in responders endorsing “agree” or “strongly agree” for a particular option and “disagree” or “strongly disagree”. Descriptive statistical analyses were conducted via SPSS 16 [27].

## Results

### Participants

Of the 39 participants who visited the online consent page, 38 agreed to participate. Three individuals consented but provided no further information and four individuals provided only demographic information. Participants completed the survey in an average of 20.5 minutes (SD 25.98). Most participants were female, had received medical help for a suicide attempt or self-injury during their lifetime, and were under 64 years old (Table 1).

**Table 1:** Demographics and previous treatment for self-harm (n=34).

Characteristics		n (%)
**Age, years**		
	<30	16 (47.1)
	30-64	16 (47.1)
	>65	2 (5.9)
**Gender**		
	Female	23 (67.6)
	Male	11 (32.4)
**Receiving medical help for suicide attempt/self-injury (lifetime)**		
	Yes	22 (64.7)
	No	12 (35.3)

### Invitation Message Subject Line Preferences

**Figure 1:**
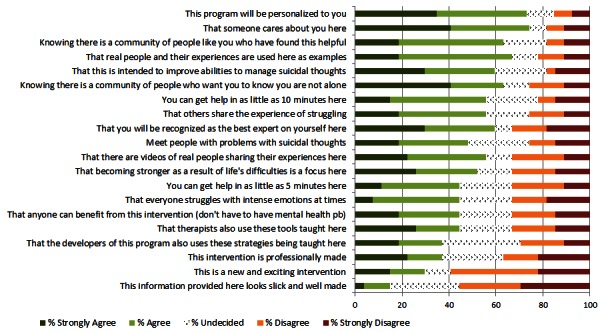
“I would like to open a message with Subject Line…”.

Of the 26 different subject lines proposed, 5 emerged as agreeable to more than 45% of responders and disagreeable to less than 25%—“Checking In”, “Touching Base”, “Between Visits Resources”, “Can We Help?”, and “Invitation to Online Support Program”. Messages advertising support as “free” or “quick” (including words as “instant”, “free”, “simple”) were less appealing to participants (Figure 1).

### Intervention Characteristic Preferences

**Figure 2:**
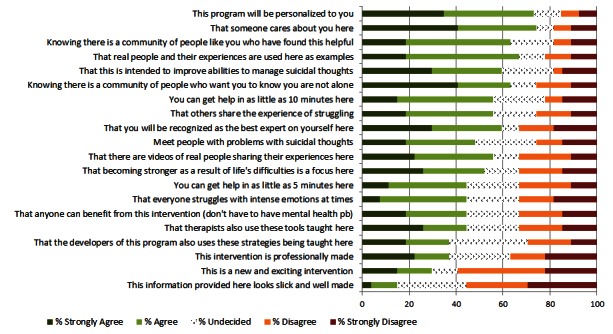
Intervention content preferences.

Twenty intervention characteristics were provided, and an average of 26.5 participants answered each question (Figure 2). The most preferred characteristics were that the program was personalized, that someone who cares personally is involved, that others have found this program helpful, that real people have found the program helpful, and that “you are not alone”. Participants least preferred a “slick” looking and “well made” program.

### Intervention Name Brand Preferences

**Figure 3:**
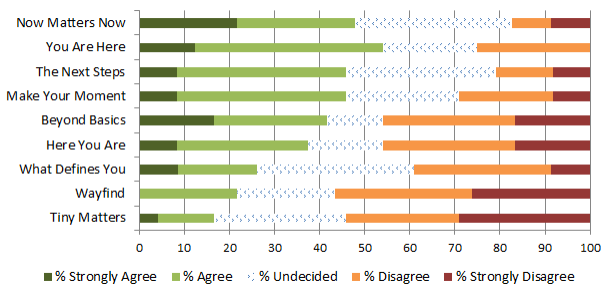
Intervention name brand preferences.

The two most preferred options for the intervention name brand were “Now Matters Now” and “You Are Here”. Preference for intervention name varied widely with an agree/disagree gap between –37.5% (“Tiny Matters”) and 30.4% (“Now Matters Now”). Each question was answered on average by 23.5 responders. See Figure 3.

### Video Evaluation

**Figure 4:**
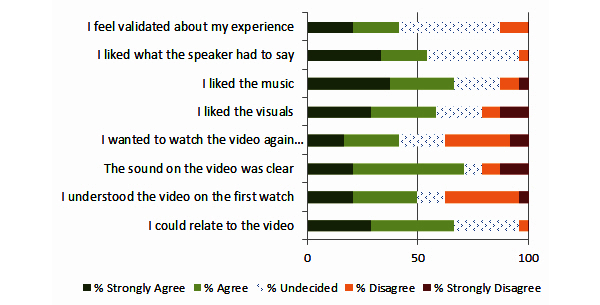
Feedback on advertisement video.

Survey participants were asked to watch a 2-minute video about the intervention and answer a series of 8 follow-up questions (see Figure 4). The purpose of the video evaluation was to identify specific aspects of the video that might need improvement. Participants were asked about technical characteristics (eg, quality of sound) and artistic impression (eg, music, visuals) as well as content itself (eg, liking what the speaker had to say).

## Discussion

### Principal Findings

The main goal of this project was to survey patients with current suicidal ideation about acceptable engagement messaging for an online intervention for individuals with suicidal thoughts. Invitation subject lines, intervention descriptions, project names, and an introductory video were reviewed. The most agreeable subject lines for invitation messages were simple inquiries about how the respondent was doing (“Checking in” and “Touching base”). Also agreeable were messages highlighting additional “resources” that “your provider thought would be helpful”. Certain messaging appeared less agreeable to participants, such as messages that proposed something “free” or “instant”. Intervention characteristics that respondents endorsed were personalization, caring, and real people with personal examples. Less important characteristics were appearance and something “new and exciting”. Two brand names relating to the present moment, “Now Matters Now” and “You Are Here”, were rated most agreeable. Overall, most respondents agreed that they could relate to the video introduction, but responses also indicated room for improvement with technical aspects and video comprehension. This project accomplished the goal of giving researchers a good place to start with messaging designed to promote uptake and engagement in an online intervention. Perhaps, more importantly, we now have a better idea about messaging options to avoid.

### Limitations

There are limitations to this project. The sample size was small and demographically narrow and thus did not allow for subgroup analyses or comparisons. However, the study sample demographics were similar to those who most commonly attempt suicide in our health care setting [25]. Individuals in the United States most likely to die by suicide, that is, males in their middle and late years, are not represented here. Future research should apply such branding and messaging questions to this specific population, but also a much larger sample in general, given the large deviations in preferences for intervention name/brand. We chose not to advertise more broadly for this survey (on websites or with advertising, which may have resulted in a larger sample) because we were interested specifically in the population our intervention is targeted to—those at increased risk for suicide attempt in the following year in our health care setting [25].

To preserve the anonymity of responders we asked only basic demographic questions. We do not know how many patients were potentially eligible to participate, so we cannot determine the response rate. Participants were informed of inclusion criteria and then were self-selected into the study, which means there may be potential selection bias. We recruited a treatment-seeking population from a mental-health clinic waiting room, and the target population of the future intervention also includes patients receiving only primary care treatment who may have different messaging preferences. We focused on the brevity of the survey, which limited our ability to explore systematic testing or manipulation of messaging content. An alternative explanation for the findings may be that the messages containing “instant/free/simple” were too vague, rather than the terms themselves being unappealing. Last, the survey options were not based on a theoretical foundation or prior hypotheses due to a lack of prior research targeting suicidal individuals for online interventions.

### Future Research

Future research should include systematic manipulation of variations around messaging (eg, comparing “Between visits resources” with “Between visits free resources”) in the hope of providing greater understanding of the effectiveness of the various message components. Future research including larger samples and such manipulations could allow us to determine how interventions might be delivered differently for subgroups of the target population (depending on symptoms, preferences, etc).

### Conclusions

We consulted with a group of patients with self-reported suicidal thoughts and past attempts to help generate the options we presented to patients. Patients with mental illnesses and suicidal ideation or behavior often report discrimination on many levels (personal, community, institutional, etc) [28]. Receiving feedback from our patient consults was particularly important since we wanted to avoid messaging that would reinforce the stigma of mental health illnesses and prevent or discourage people affected by mental illness from seeking treatment. This paper provides one model (including development of an engagement survey, audience for an engagement survey, methods for presenting results of an engagement survey) for including target users in the development of uptake strategies for online mental health interventions. Finally, large pragmatic clinical trials should be conducted in order to identify whether an online intervention could reduce suicide attempts or deaths on a large scale.

## Multimedia Appendix 1

"Your Feedback Counts"- survey with logos included.

## Multimedia Appendix 2

Full survey results.

## Abbreviations

PHQ: Patient Health Questionnaire
