# Figure Correction: Designing Messaging to Engage Patients in an Online Suicide Prevention Intervention: Survey Results From Patients With Current Suicidal Ideation

**DOI:** 10.2196/jmir.4412

**Published:** 2015-04-13

**Authors:** Ursula Whiteside, Anita Lungu, Julie Richards, Gregory E Simon, Sarah Clingan, Jaeden Siler, Lorilei Snyder, Evette Ludman

**Affiliations:** ^1^Group Health Research InstituteSeattle, WAUnited States

The authors of “Designing Messaging to Engage Patients in an Online Suicide Prevention Intervention: Survey Results From Patients With Current Suicidal Ideation” (http://www.jmir.org/2014/2/e42/) have, during the final proofreading process, inadvertently added the same image file for Figures 1 and 2. Figure 1 has now been updated with the correct image, with the caption “I would like to open a message with Subject Line...”. This error has been corrected in the online version of the paper on the JMIR website on April 13, 2015, together with publishing this correction notice. A correction notice has been sent to PubMed and the correct full-text has been resubmitted to Pubmed Central and other full-text repositories.

**Figure 1:**
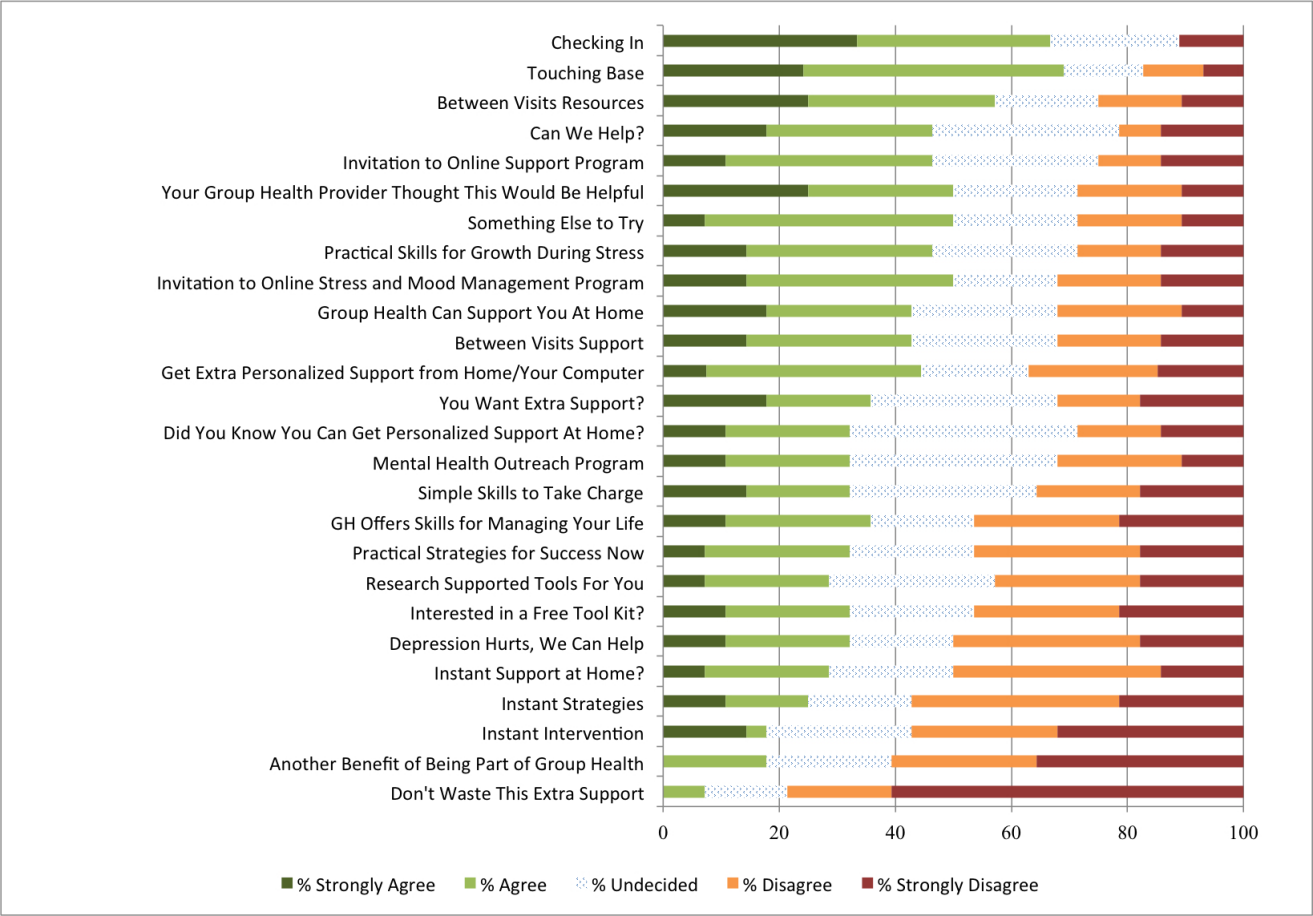
“I would like to open a message with Subject Line...”.

