# Supplementary material for: Designing Messaging to Engage Patients in an Online Suicide Prevention Intervention: Survey Results From Patients With Current Suicidal Ideation
Source: J Med Internet Res. 2014 Feb 7;16(2):e42. doi: 10.2196/jmir.3173 (PMC3936268; doi:10.2196/jmir.3173)
Supplement: Supplementary file 1 [file jmir_v16i2e42_app1.pdf]

Group Health Research Institute

Your Feedback Counts:

An Online Study

0%

Consent Form

Group Health Research Institute Researchers

Ursula Whiteside, PhD, Lead Researcher
Gregory Simon, MD, MPH, Anita Lungu, PhD, and Evette Ludman PhD, Co-Investigators
Tara Beatty and Belinda Operskalski, Project Managers
(All investigators can be left a message at 1-888-324-3166)

We are in the process of creating materials for Online Interventions. To improve our materials we are asking people to tell us what they think about these materials as we develop them. This is what the current feedback study is all about. This form explains the details further.

You do not have to be in this feedback study unless you want to. Even if you say yes to being part of this study, you can quit at any time. Your choice will not affect your care or benefits at Group Health in any way. If after reading this information you decide to join, you must click "**I AGREE TO PARTICIPATE IN THIS FEEDBACK RESEARCH**" at the bottom of this page.

What is this study about?

We are developing Online Interventions to help people who think about suicide. Right now, we are developing the materials for these interventions that we plan to post online so people can use them on a computer. These Online Interventions are under development anc we are asking you to help us by giving us your opinion about the materials we create. This feedback study is not intended to help you personally. At the same time, by accepting to be part of this study you would be helping us improve these Online Interventions for people who think about suicide.

What will happen if I take part in this study?

You are eligible to be part of this current feedback study if you have regularly been thinking about suicide (i.e., more than half the days o nearly every day recently).
If you join the study, we may ask you to:

- **Review short videos and other written materials we are developing.**
- **Complete an online survey.** This survey will ask what you think about pieces of the online materials that you reviewed. The

survey will take 5 – 30 minutes to fill out, depending on how long you decide to review the content. You can skip any question yo want to.

You can choose to complete the survey anonymously, or if you provide your name and address at the end of this survey we will mail you $10 in cash or a gift card for $10 as a thank you.

Will being in this study help me?

This current feedback will not help you personally, but we hope the results of this study will help improve care for people who regularly think about suicide.
We do not know whether or not you will be invited to join future studies about this type of research and will not track your information for that purpose.

Can anything bad happen to me from being in this study?

You might feel uncomfortable having your answers recorded. However, you may skip any questions you don't want to answer.
If you provide your name and address, it is possible that someone other than the researchers could find out you were in the study. The steps we take to keep this from happening are described below.

How will you protect my confidentiality?

All Group Health researchers sign a confidentiality pledge that requires them to keep your information private.
The researchers listed on the first page and their staff will use your study information for research only. Your name and address, if you choose to provide them, will not be linked to the opinions you give us as part of this study. *We won't tell your doctor whether or not you join this study or add information to your medical record.*
If you provide your name and contact information at the end of this survey, we plan to keep your study information as described in this form until you have been mailed a $10 in cash or gift card, and not longer than 10/01/2013. At that time, we will destroy any study recorc that include your name and address.

Who do I call if I have questions about being in this study?

If you have questions about the research, you can call our confidential message center (1-888-324-3166). If you have questions about your rights as a research participant, you can call (206)287-2919.
Please print this page for your records.

Thank you for considering to be part of this study and to help us with your feedback on materials.

MY DECISION ABOUT THIS STUDY

- ○ **I AGREE TO PARTICIPATE IN THIS RESEARCH STUDY:** I have read the information above and I have called the researchers to ask any questions I may have. I know I may leave the study at any time. I volunteer to participate and give permission to use my feedback to improve the materials being developed.
- ○ **I DO NOT WISH TO PARTICIPATE IN THIS RESEARCH**

These questions will help us understand how different groups of people respond to our questions.

Which age group are you in?

- ○ Under 30 years of age
- ○ 30 to 64 years of age
- ○ 65 years of age or older

Are you male or female?

- ○ Male
- ○ Female

Have you ever received medical treatment (e.g., at a urgent care, ER, hospital, or clinic) for a suicide attempt or another type of intentional self injury?

- ○ No
- ○ Yes

You did not answer one or more of the questions on the last page. If this was not intentional, please go back and respond.

Continued, these questions will help us understand how different groups of people respond to our questions.

Have you used MyGroupHealth (www.ghc.org), the website or mobile phone application, to **Send Messages** to Group Health providers?
Below is an image showing where that access is located within the group health website.

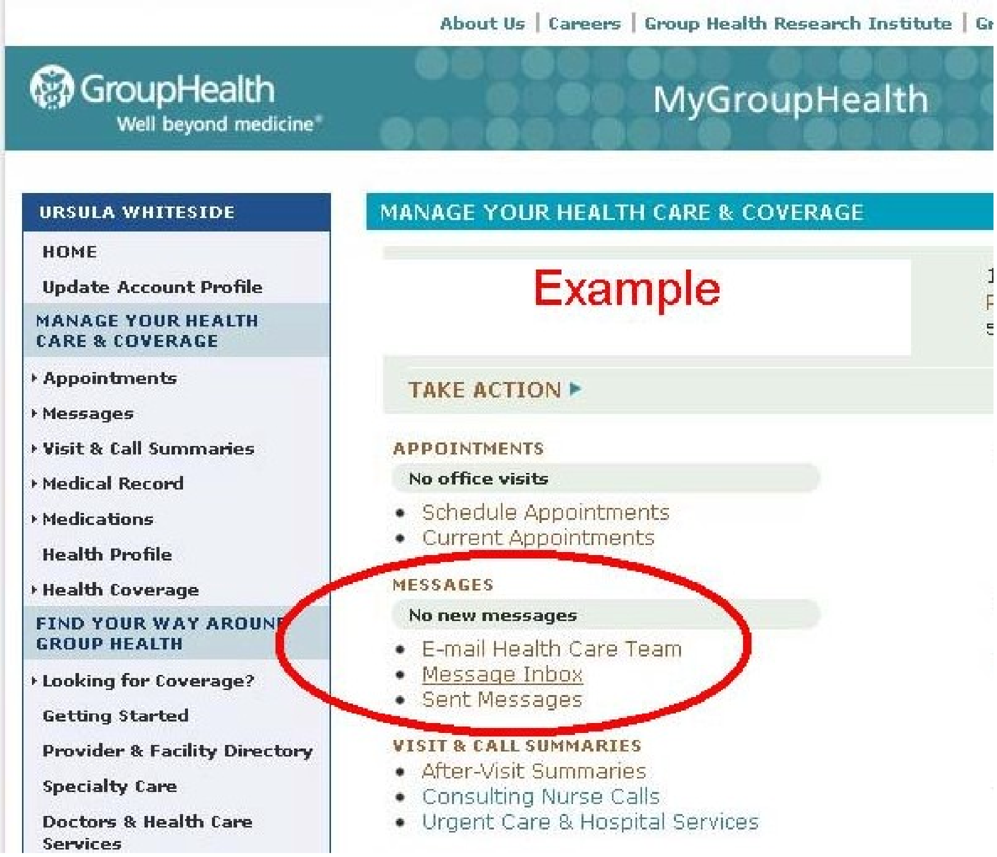

- ○ No, I have never used MyGroupHealth to send messages to Group Health Providers
- ○ Yes, but probably NOT in past six months
- ○ Yes, and probably DID in the past six months

▶ We have reason to believe that some people may no longer be receiving email alerts when they have a new message in MyGroupHealth.

Do you remember ever receiving email alerts when you have a new secure message from MyGroupHealth?

These alerts come from the email address MyGroupHealth@ghc.org.

- ○ No, I don't think I've received such alerts
- ○ Yes, but I don't recall receiving a reminder lately
- ○ Yes, I think I've received one in the past few months

▶ Do you receive these alerts at a Gmail address, a Hotmail address, or another address?

- ○ Yes, at a Gmail email address
- ○ Yes, at a Hotmail email address
- ○ No, neither a Gmail or Hotmail email address
- ○ I'm not sure.

You did not answer one or more of the questions on the last page. If this was not intentional, please go back and respond.

Imagine the following scenario

Assume for a moment that you recently saw your provider at Group Health and shared on a depression assessment that you had been having thoughts that you would be better off dead, or of hurting yourself in some way. About a week later you receive a secure email message in your MyGroupHealth message inbox.

We are interested in your thoughts about which Subject Line of that secure message would be most effective in getting people to open that message.

Based on this scenario, please answer the following questions to help us determine which of the following message Subject Lines to use to invite people to our online intervention.

It is most helpful if you can provide a range of responses as opposed to classifying all options with the same rating.

The image below gives an example of where the subject line would show up within the MyGroupHealth online portal.

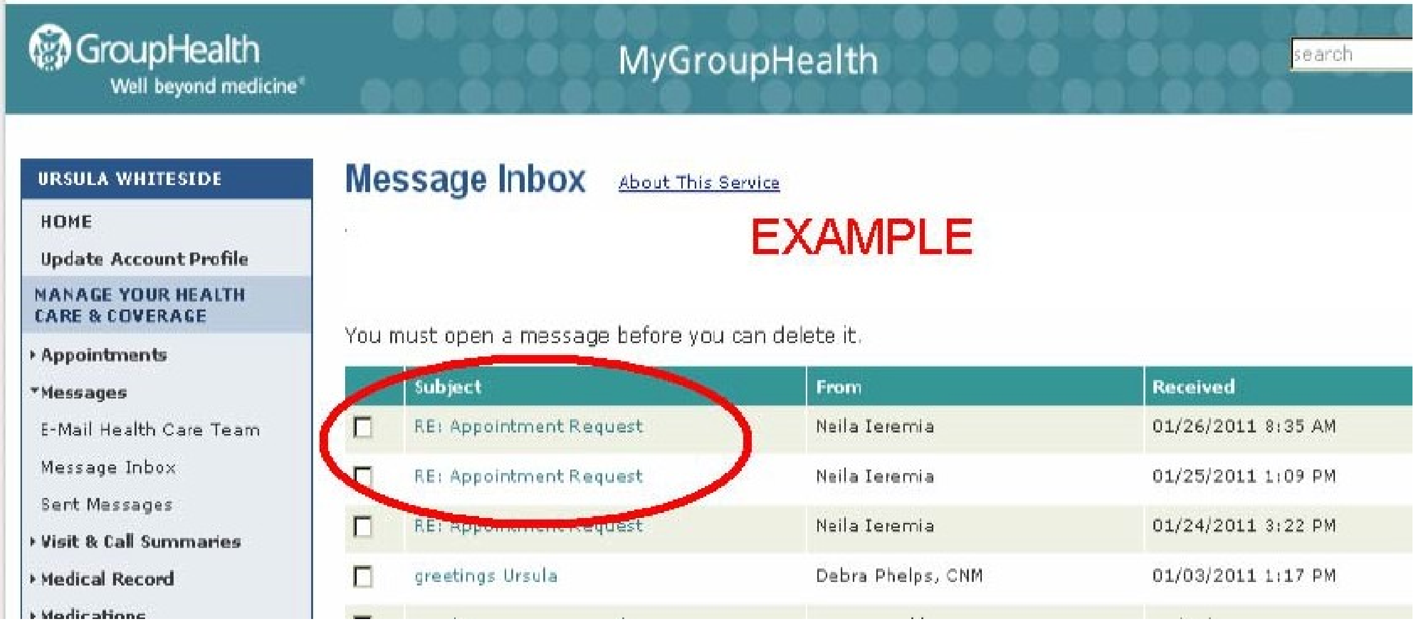

**I would want to open a message with this Subject Line...**	Strongly Disagree	Disagree	Undecided	Agree	Strongly Agree
Another Benefit of Being Part of Group Health	○	○	○	○	○
Research Supported Tools For You	○	○	○	○	○
Can We Help?	○	○	○	○	○
You Want Extra Support?	○	○	○	○	○
Invitation to Online Support Program	○	○	○	○	○
Something Else to Try	○	○	○	○	○
Don't Waste This Extra Support	○	○	○	○	○
Practical Skills for Growth During Stress	○	○	○	○	○
Simple Skills to Take Charge	○	○	○	○	○
Between Visits Support	○	○	○	○	○
	Strongly Disagree	Disagree	Undecided	Agree	Strongly Agree
Practical Strategies for Success Now	○	○	○	○	○
Depression Hurts, We Can Help	○	○	○	○	○
Group Health Can Support You At Home	○	○	○	○	○
Touching Base	○	○	○	○	○
Invitation to Online Stress and Mood Management Program	○	○	○	○	○
Instant Strategies	○	○	○	○	○
GH Offers Skills for Managing Your Life	○	○	○	○	○
Your Group Health Provider Thought This Would Be Helpful	○	○	○	○	○
Between Visits Resources	○	○	○	○	○
Interested in a Free Tool Kit?	○	○	○	○	○
	Strongly Disagree	Disagree	Undecided	Agree	Strongly Agree
Instant Intervention					

	○	○	○	○	○
Instant Support at Home?	○	○	○	○	○
Get Extra Personalized Support from Home/Your Computer	○	○	○	○	○
Did You Know You Can Get Personalized Support At Home?	○	○	○	○	○
Mental Health Outreach Program	○	○	○	○	○
Checking In	○	○	○	○	○
Something Else to Try	○	○	○	○	○

What **Subject Line** would you recommend we use?
You can make up your own or copy one from above.

Please note that responses are not reviewed in a timely manner. The experience of suicidal thoughts is not an uncommon part of the human experience. If you need support now, you can call the King County Crisis Line 866-4-CRISIS 866-427-4747), The National Suicide Prevention Lifeline (1-800-273-8255), or a your Group Health Consulting Nurse (1-800-297-6877) or go to your nearest emergency room or call 911.

You did not answer one or more of the questions on the last page. If this was not intentional, please go back and respond.

Continuing with the same Scenario

Assume for a moment that you recently saw your provider at Group Health and shared on a depression assessment that you had been having thoughts that you would be better off dead, or of hurting yourself in some way on more than half of the days in the past few weeks. About a week later you receive a secure email message in your MyGroupHealth message inbox.

Answer the following questions to help us determine which of the following topics would lead you to visit an Online Intervention.

It is most helpful if you can provide a range of responses.

**Knowing this would get me to visit an Online Intervention...**	Strongly Disagree	Disagree	Undecided	Agree	Strongly Agree
Knowing there is a community of people who want you to know you are not alone	○	○	○	○	○
That there are videos of real people sharing their experiences here	○	○	○	○	○
That real people and their experiences are used here as examples	○	○	○	○	○
That the developers of this program also uses these strategies being taught here	○	○	○	○	○
This intervention is professionally made	○	○	○	○	○
That someone cares about you here	○	○	○	○	○
That therapists also use these tools taught here	○	○	○	○	○
You can get help in as little as 10 minutes here	○	○	○	○	○
Knowing there is a community of people like you who have found this helpful	○	○	○	○	○
This is a new and exciting intervention	○	○	○	○	○
	Strongly Disagree	Disagree	Undecided	Agree	Strongly Agree
That others share the experience of struggling	○	○	○	○	○
That becoming stronger as a result of life's difficulties is a focus here	○	○	○	○	○
That you will be recognized as the best expert on yourself here	○	○	○	○	○

That everyone struggles with intense emotions at times					
This information provided here looks slick and well made					
Meet people with problems with suicidal thoughts					
That anyone can benefit from this intervention (that you don't have to have a mental health problem)					
You can get help in as little as 5 minutes here					
This program will be personalized to you					
That this is intended to improve abilities to manage suicidal thoughts					

What do you think would get people to visit an **Online Intervention**?
You can make up your own or copy one from above

Please note that responses are not reviewed in a timely manner. The experience of suicidal thoughts is not an uncommon part of the human experience. If you need support now, you can call the King County Crisis Line 866-4-CRISIS 866-427-4747), The National Suicide Prevention Lifeline (1-800-273-8255), or a your Group Health Consulting Nurse (1-800-297-6877) or go to your nearest emergency room or call 911.

You did not answer one or more of the questions on the last page. If this was not intentional, please go back and respond.

Please watch this short video.

This video includes sound, so you may need headphones if you are not in a private area.

Test video below:

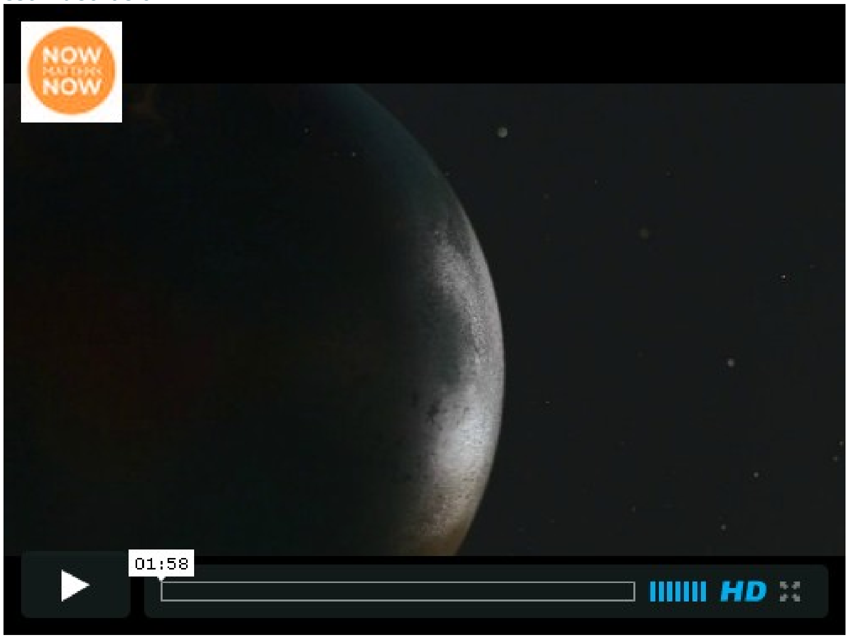

General Thoughts about the Video

Please note that responses are not reviewed in a timely manner. The experience of suicidal thoughts is not an uncommon part of the human experience. If you need support now, you can call the King County Crisis Line 866-4-CRISIS 866-427-4747), The National Suicide Prevention Lifeline (1-800-273-8255), or a your Group Health Consulting Nurse (1-800-297-6877) or go to your nearest emergency room or call 911.

**Please rate the Video...**	Strongly Disagree	Disagree	Undecided	Agree	Strongly Agree	Video Didn't Work

I wanted to watch the video again because I was interested	◯	◯	◯	◯	◯	◯
I understood the video on the first watch	◯	◯	◯	◯	◯	◯
I liked the music	◯	◯	◯	◯	◯	◯
I liked what the speaker had to say	◯	◯	◯	◯	◯	◯
I could relate to the video	◯	◯	◯	◯	◯	◯
I liked the visuals	◯	◯	◯	◯	◯	◯
I feel validated about my experience	◯	◯	◯	◯	◯	◯
The sound on the video was clear	◯	◯	◯	◯	◯	◯

You did not answer one or more of the questions on the last page. If this was not intentional, please go back and respond.

We are working on coming up with a name for our project. It is based on a well-known treatment called Dialectical Behavioral Therapy. This intervention is not Dialectical Behavioral Therapy because that is a very intensive in-person intensive treatment. Can you rate the following names on likability?

We have a wonderful group of people who we are consulting with us as part of this intervention development (Team SPR). These include people with varying experiences with suicidal thoughts and behaviors. We have come up with a list of possible names for this intervention.

When reviewing this list, please keep in mind that the intervention is focused on teaching people strategies that they can try immediately. They are also focused a foundation of learning to focus on the current moment (aka mindful attention or mindfulness). Can you tell us which ones you prefer and fit best based on our description?	Strongly Disagree	Disagree	Undecided	Agree	Strongly Agree
Here You Are	◯	◯	◯	◯	◯
Make Your Moment	◯	◯	◯	◯	◯
Now Matters Now	◯	◯	◯	◯	◯
Wayfind	◯	◯	◯	◯	◯
Tiny Matters	◯	◯	◯	◯	◯
Beyond Basics	◯	◯	◯	◯	◯
The Next Steps	◯	◯	◯	◯	◯
What Defines You	◯	◯	◯	◯	◯
You Are Here	◯	◯	◯	◯	◯

NOWMATTERSNOW

NOW MATTERS NOW

Which do you prefer of the above logo styles?

- ○ Rectangle Version
- ◉ Circle Version

Thank you for considering being a part of this project.

Powered by DatStat
