# Supplementary material for: Designing Messaging to Engage Patients in an Online Suicide Prevention Intervention: Survey Results From Patients With Current Suicidal Ideation
Source: J Med Internet Res. 2014 Feb 7;16(2):e42. doi: 10.2196/jmir.3173 (PMC3936268; doi:10.2196/jmir.3173)
Supplement: Supplementary file 2 [file jmir_v16i2e42_app2.pdf]

CONSENTRESPONSE: MY DECISION ABOUT THIS STUDY		
**Prompt**	**Count**	**Pct**
**I AGREE TO PARTICIPATE IN THIS RESEARCH STUDY: I have read the information above and I have called the researchers to ask any questions I may have. I know I may leave the study at any time. I volunteer to participate and give permission to use my feedback to improve the materials being developed.**	**37**	**97.37%**
I DO NOT WISH TO PARTICIPATE IN THIS RESEARCH	1	2.63%
**Aggregates**		
Count		38
Min		0
Max		1
Sum		37
Mean		0.97
Std Dev		0.16
Variance		0.03

AGEGP: Which age group are you in?		
**Prompt**	**Count**	**Pct**
Under 30 years of age	15	45.45%
**30 to 64 years of age**	**16**	**48.48%**
65 years of age or older	2	6.06%
**Aggregates**		
Count		33
Min		1
Max		3
Sum		53
Mean		1.61
Std Dev		0.61
Variance		0.37

SEX: Are you male or female?		
**Prompt**	**Count**	**Pct**
Male	10	30.30%
**Female**	**23**	**69.70%**
**Aggregates**		
Count		33
Min		0
Max		1
Sum		23
Mean		0.7
Std Dev		0.47
Variance		0.22

SELFINJHX: Have you ever received medical treatment (e.g., at a urgent care, ER, hospital, or clinic) for a suicide attempt or another type of intentional self injury?		
**Prompt**	**Count**	**Pct**
No	11	33.33%
**Yes**	**22**	**66.67%**
**Aggregates**		
Count		33
Min		0
Max		1
Sum		22

Mean	0.67
Std Dev	0.48
Variance	0.23

MYGPHEALTH: Have you used MyGroupHealth (www.ghc.org), the website or mobile phone application, to Send Messages to Group Health providers? Below is an image showing where that access is located within the group health website.

Prompt	Count	Pct
No, I have never used MyGroupHealth to send messages to Group Health Providers	3	9.68%
Yes, but probably NOT in past six months	4	12.90%
**Yes, and probably DID in the past six months**	**24**	**77.42%**
**Aggregates**		
Count		31
Min		0
Max		2
Sum		52
Mean		1.68
Std Dev		0.65
Variance		0.43

ALERTREMEMBER: We have reason to believe that some people may no longer be receiving email alerts when they have a new message in MyGroupHealth. Do you remember ever receiving email alerts when you have a new secure message from MyGroupHealth? These alerts come from the email address MyGroupHealth@ghc.org.

Prompt	Count	Pct
No, I don't think I've received such alerts	6	19.35%
Yes, but I don't recall receiving a reminder lately	6	19.35%
**Yes, I think I've received one in the past few months**	**19**	**61.29%**
**Aggregates**		
Count		31
Min		0
Max		2
Sum		44
Mean		1.42
Std Dev		0.81
Variance		0.65

ALERTEMAILTYPE: Do you receive these alerts at a Gmail address, a Hotmail address, or another address?

Prompt	Count	Pct
**Yes, at a Gmail email address**	**14**	**45.16%**
Yes, at a Hotmail email address	4	12.90%
No, neither a Gmail or Hotmail email address	7	22.58%
I'm not sure.	6	19.35%
**Aggregates**		
Count		31
Min		0
Max		3
Sum		36
Mean		1.16
Std Dev		1.21
Variance		1.47

Q1: Did You Know You Can Get Personalized Support At Home?

Prompt	Count	Pct
Strongly Disagree	4	14.81%
Disagree	4	14.81%
**Undecided**	**11**	**40.74%**
Agree	5	18.52%
Strongly Agree	3	11.11%
**Aggregates**		
Count		27
Min		0
Max		4
Sum		53
Mean		1.96
Std Dev		1.19
Variance		1.42

Q2: Instant Support at Home?

Prompt	Count	Pct
Strongly Disagree	4	14.81%
**Disagree**	**9**	**33.33%**
Undecided	6	22.22%
Agree	6	22.22%
Strongly Agree	2	7.41%
**Aggregates**		
Count		27
Min		0
Max		4
Sum		47
Mean		1.74
Std Dev		1.2
Variance		1.43

Q3: Instant Strategies

Prompt	Count	Pct
Strongly Disagree	5	18.52%
**Disagree**	**10**	**37.04%**
Undecided	5	18.52%
Agree	4	14.81%
Strongly Agree	3	11.11%
**Aggregates**		
Count		27
Min		0
Max		4
Sum		44
Mean		1.63
Std Dev		1.28
Variance		1.63

Q4: Group Health Can Support You At Home

Prompt	Count	Pct
Strongly Disagree	3	11.11%
Disagree	5	18.52%

**Undecided**	**7**	**25.93%**
**Agree**	**7**	**25.93%**
Strongly Agree	5	18.52%
**Aggregates**		
Count		27
Min		0
Max		4
Sum		60
Mean		2.22
Std Dev		1.28
Variance		1.64

Q5: Get Extra Personalized Support from Home/Your Computer

Prompt	Count	Pct
Strongly Disagree	4	15.38%
Disagree	6	23.08%
Undecided	4	15.38%
**Agree**	**10**	**38.46%**
Strongly Agree	2	7.69%
**Aggregates**		
Count		26
Min		0
Max		4
Sum		52
Mean		2
Std Dev		1.26
Variance		1.6

Q6: GH Offers Skills for Managing Your Life

Prompt	Count	Pct
Strongly Disagree	6	22.22%
**Disagree**	**7**	**25.93%**
Undecided	4	14.81%
**Agree**	**7**	**25.93%**
Strongly Agree	3	11.11%
**Aggregates**		
Count		27
Min		0
Max		4
Sum		48
Mean		1.78
Std Dev		1.37
Variance		1.87

Q7: You Want Extra Support?

Prompt	Count	Pct
Strongly Disagree	4	14.81%
Disagree	4	14.81%
**Undecided**	**9**	**33.33%**
Agree	5	18.52%
Strongly Agree	5	18.52%
**Aggregates**		
Count		27

Min	0
Max	4
Sum	57
Mean	2.11
Std Dev	1.31
Variance	1.72

Q8: Depression Hurts, We Can Help		
**Prompt**	**Count**	**Pct**
Strongly Disagree	5	18.52%
**Disagree**	**8**	**29.63%**
Undecided	5	18.52%
Agree	6	22.22%
Strongly Agree	3	11.11%
**Aggregates**		
Count		27
Min		0
Max		4
Sum		48
Mean		1.78
Std Dev		1.31
Variance		1.72

Q9: Can We Help?		
**Prompt**	**Count**	**Pct**
Strongly Disagree	4	14.81%
Disagree	2	7.41%
**Undecided**	**9**	**33.33%**
Agree	7	25.93%
Strongly Agree	5	18.52%
**Aggregates**		
Count		27
Min		0
Max		4
Sum		61
Mean		2.26
Std Dev		1.29
Variance		1.66

Q10: Another Benefit of Being Part of Group Health		
**Prompt**	**Count**	**Pct**
**Strongly Disagree**	**10**	**37.04%**
Disagree	7	25.93%
Undecided	6	22.22%
Agree	4	14.81%
Strongly Agree	0	.00%
**Aggregates**		
Count		27
Min		0
Max		3
Sum		31
Mean		1.15

Std Dev	1.1
Variance	1.21

Q11: Don't Waste This Extra Support

Prompt	Count	Pct
**Strongly Disagree**	**16**	**59.26%**
Disagree	5	18.52%
Undecided	4	14.81%
Agree	2	7.41%
Strongly Agree	0	.00%
**Aggregates**		
Count		27
Min		0
Max		3
Sum		19
Mean		0.7
Std Dev		0.99
Variance		0.99

Q12: Your Group Health Provider Thought This Would Be Helpful

Prompt	Count	Pct
Strongly Disagree	3	11.11%
Disagree	5	18.52%
Undecided	6	22.22%
**Agree**	**7**	**25.93%**
Strongly Agree	6	22.22%
**Aggregates**		
Count		27
Min		0
Max		4
Sum		62
Mean		2.3
Std Dev		1.32
Variance		1.75

Q13: Checking In

Prompt	Count	Pct
Strongly Disagree	3	11.54%
Disagree	0	.00%
Undecided	6	23.08%
**Agree**	**9**	**34.62%**
Strongly Agree	8	30.77%
**Aggregates**		
Count		26
Min		0
Max		4
Sum		71
Mean		2.73
Std Dev		1.25
Variance		1.56

Q14: Touching Base

Prompt	Count	Pct
Strongly Disagree	2	7.14%
Disagree	3	10.71%
Undecided	4	14.29%
**Agree**	**12**	**42.86%**
Strongly Agree	7	25.00%
**Aggregates**		
Count		28
Min		0
Max		4
Sum		75
Mean		2.68
Std Dev		1.19
Variance		1.41

Q15: Something Else to Try		
**Prompt**	**Count**	**Pct**
Strongly Disagree	3	11.11%
Disagree	4	14.81%
Undecided	6	22.22%
**Agree**	**12**	**44.44%**
Strongly Agree	2	7.41%
**Aggregates**		
Count		27
Min		0
Max		4
Sum		60
Mean		2.22
Std Dev		1.15
Variance		1.33

Q16: Between Visits Support		
**Prompt**	**Count**	**Pct**
Strongly Disagree	4	14.81%
Disagree	5	18.52%
**Undecided**	**7**	**25.93%**
**Agree**	**7**	**25.93%**
Strongly Agree	4	14.81%
**Aggregates**		
Count		27
Min		0
Max		4
Sum		56
Mean		2.07
Std Dev		1.3
Variance		1.69

Q17: Between Visits Resources		
**Prompt**	**Count**	**Pct**
Strongly Disagree	3	11.11%
Disagree	4	14.81%
Undecided	5	18.52%
**Agree**	**8**	**29.63%**

Strongly Agree	7	25.93%
**Aggregates**		
Count		27
Min		0
Max		4
Sum		66
Mean		2.44
Std Dev		1.34
Variance		1.79

Q18: Something Else to Try

Prompt	Count	Pct
Strongly Disagree	5	18.52%
Disagree	5	18.52%
**Undecided**	**7**	**25.93%**
Agree	6	22.22%
Strongly Agree	4	14.81%
**Aggregates**		
Count		27
Min		0
Max		4
Sum		53
Mean		1.96
Std Dev		1.34
Variance		1.81

Q19: Interested in a Free Tool Kit?

Prompt	Count	Pct
Strongly Disagree	5	18.52%
**Disagree**	**7**	**25.93%**
Undecided	6	22.22%
Agree	6	22.22%
Strongly Agree	3	11.11%
**Aggregates**		
Count		27
Min		0
Max		4
Sum		49
Mean		1.81
Std Dev		1.3
Variance		1.7

Q20: Simple Skills to Take Charge

Prompt	Count	Pct
Strongly Disagree	5	18.52%
Disagree	5	18.52%
**Undecided**	**9**	**33.33%**
Agree	4	14.81%
Strongly Agree	4	14.81%
**Aggregates**		
Count		27
Min		0

Max	4
Sum	51
Mean	1.89
Std Dev	1.31
Variance	1.72

Q21: Practical Skills for Growth During Stress

Prompt	Count	Pct
Strongly Disagree	4	14.81%
Disagree	4	14.81%
Undecided	6	22.22%
**Agree**	**9**	**33.33%**
Strongly Agree	4	14.81%
**Aggregates**		
Count		27
Min		0
Max		4
Sum		59
Mean		2.19
Std Dev		1.3
Variance		1.7

Q22: Research Supported Tools For You

Prompt	Count	Pct
Strongly Disagree	5	18.52%
Disagree	6	22.22%
**Undecided**	**8**	**29.63%**
Agree	6	22.22%
Strongly Agree	2	7.41%
**Aggregates**		
Count		27
Min		0
Max		4
Sum		48
Mean		1.78
Std Dev		1.22
Variance		1.49

Q23: Practical Strategies for Success Now

Prompt	Count	Pct
Strongly Disagree	5	18.52%
**Disagree**	**7**	**25.93%**
Undecided	6	22.22%
**Agree**	**7**	**25.93%**
Strongly Agree	2	7.41%
**Aggregates**		
Count		27
Min		0
Max		4
Sum		48
Mean		1.78
Std Dev		1.25
Variance		1.56

Q24: Instant Intervention		
**Prompt**	**Count**	**Pct**
**Strongly Disagree**	**8**	**29.63%**
Disagree	7	25.93%
Undecided	7	25.93%
Agree	1	3.70%
Strongly Agree	4	14.81%
**Aggregates**		
Count		27
Min		0
Max		4
Sum		40
Mean		1.48
Std Dev		1.37
Variance		1.87

Q25: Mental Health Outreach Program		
**Prompt**	**Count**	**Pct**
Strongly Disagree	3	11.11%
Disagree	6	22.22%
**Undecided**	**10**	**37.04%**
Agree	5	18.52%
Strongly Agree	3	11.11%
**Aggregates**		
Count		27
Min		0
Max		4
Sum		53
Mean		1.96
Std Dev		1.16
Variance		1.34

Q28: Invitation to Online Support Program		
**Prompt**	**Count**	**Pct**
Strongly Disagree	4	14.81%
Disagree	3	11.11%
Undecided	7	25.93%
**Agree**	**10**	**37.04%**
Strongly Agree	3	11.11%
**Aggregates**		
Count		27
Min		0
Max		4
Sum		59
Mean		2.19
Std Dev		1.24
Variance		1.54

Q29: Invitation to Online Stress and Mood Management Program		
**Prompt**	**Count**	**Pct**
Strongly Disagree	4	14.81%

Disagree	4	14.81%
Undecided	5	18.52%
**Agree**	**10**	**37.04%**
Strongly Agree	4	14.81%
**Aggregates**		
Count		27
Min		0
Max		4
Sum		60
Mean		2.22
Std Dev		1.31
Variance		1.72

SUBJECTFREETEXT: What Subject Line would you recommend we use? You can make up your own or copy one from above.

Row	Comment
1	xyk
2	What's missing?
3	We're Listening
4	Checking in
5	Your Group Health Provider thought this w ould be helpful.
6	Invitation to Online Stress and Mood Management Program
7	Touching base, how can w e help?
8	Checking in
9	Just checking in Touching base update me
10	Betw een Visits Support
11	Strategies and Support
12	"Betw een Visits Resources" and "Touching Base" feel like they could be personalized messages instead of form letters. I like that.
13	Get extra support from your computer, etc. Also, subjects that imply the provider actually cares like "checking in" are great
14	Really liked "Checking In" and "Touching Base". Feels a bit more personal/about me, yet not as revealing to others that might see the Subject Line.
15	Checking In
16	I do not use this feature enough to comment
17	Practical Skills for Grow th During Stress
18	Please take a look, this may be helpful for you.
19	Don't hurt yourself: There's help. [Healthcare Providers Name] w ants you to try this. There's at least one person that w ould miss you! Here's proof. Online support, GH at your ow n home!
20	Therapy
21	Don't w aste this extra support
22	How do you feel today?
23	Touching Base.
24	Depression hurts, w e can help
25	Staying Safe at Home/Betw een Visits
26	Get Extra Personalized Support from Home/Your Computer

Q27: That others share the experience of struggling

Prompt	Count	Pct
Strongly Disagree	3	11.54%
Disagree	4	15.38%
Undecided	5	19.23%
**Agree**	**9**	**34.62%**
Strongly Agree	5	19.23%
**Aggregates**		

Count	26
Min	0
Max	4
Sum	61
Mean	2.35
Std Dev	1.29
Variance	1.68

Q30: That this is intended to improve abilities to manage suicidal thoughts

Prompt	Count	Pct
Strongly Disagree	4	15.38%
Disagree	1	3.85%
Undecided	6	23.08%
Agree	7	26.92%
**Strongly Agree**	**8**	**30.77%**
**Aggregates**		
Count		26
Min		0
Max		4
Sum		66
Mean		2.54
Std Dev		1.39
Variance		1.94

Q31: That real people and their experiences are used here as examples

Prompt	Count	Pct
Strongly Disagree	3	11.54%
Disagree	3	11.54%
Undecided	3	11.54%
**Agree**	**12**	**46.15%**
Strongly Agree	5	19.23%
**Aggregates**		
Count		26
Min		0
Max		4
Sum		65
Mean		2.5
Std Dev		1.27
Variance		1.62

Q32: That therapists also use these tools taught here

Prompt	Count	Pct
Strongly Disagree	4	15.38%
Disagree	5	19.23%
Undecided	6	23.08%
Agree	4	15.38%
**Strongly Agree**	**7**	**26.92%**
**Aggregates**		
Count		26
Min		0
Max		4
Sum		57
Mean		2.19

Std Dev	1.44
Variance	2.08

Q33: That anyone can benefit from this intervention (that you don't have to have a mental health problem)

Prompt	Count	Pct
Strongly Disagree	4	15.38%
Disagree	5	19.23%
Undecided	6	23.08%
**Agree**	**7**	**26.92%**
Strongly Agree	4	15.38%
**Aggregates**		
Count		26
Min		0
Max		4
Sum		54
Mean		2.08
Std Dev		1.32
Variance		1.75

Q34: That you will be recognized as the best expert on yourself here

Prompt	Count	Pct
Strongly Disagree	4	15.38%
Disagree	4	15.38%
Undecided	2	7.69%
**Agree**	**8**	**30.77%**
**Strongly Agree**	**8**	**30.77%**
**Aggregates**		
Count		26
Min		0
Max		4
Sum		64
Mean		2.46
Std Dev		1.48
Variance		2.18

Q35: That everyone struggles with intense emotions at times

Prompt	Count	Pct
Strongly Disagree	5	19.23%
Disagree	4	15.38%
Undecided	6	23.08%
**Agree**	**9**	**34.62%**
Strongly Agree	2	7.69%
**Aggregates**		
Count		26
Min		0
Max		4
Sum		51
Mean		1.96
Std Dev		1.28
Variance		1.64

Q36: That someone cares about you here		
**Prompt**	**Count**	**Pct**
Strongly Disagree	3	11.54%
Disagree	1	3.85%
Undecided	2	7.69%
Agree	9	34.62%
**Strongly Agree**	**11**	**42.31%**
**Aggregates**		
Count		26
Min		0
Max		4
Sum		76
Mean		2.92
Std Dev		1.32
Variance		1.75

Q37: Knowing there is a community of people like you who have found this helpful		
**Prompt**	**Count**	**Pct**
Strongly Disagree	3	11.54%
Disagree	2	7.69%
Undecided	5	19.23%
**Agree**	**11**	**42.31%**
Strongly Agree	5	19.23%
**Aggregates**		
Count		26
Min		0
Max		4
Sum		65
Mean		2.5
Std Dev		1.24
Variance		1.54

Q38: Knowing there is a community of people who want you to know you are not alone		
**Prompt**	**Count**	**Pct**
Strongly Disagree	3	11.54%
Disagree	3	11.54%
Undecided	3	11.54%
Agree	6	23.08%
**Strongly Agree**	**11**	**42.31%**
**Aggregates**		
Count		26
Min		0
Max		4
Sum		71
Mean		2.73
Std Dev		1.43
Variance		2.04

Q39: Meet people with problems with suicidal thoughts		
**Prompt**	**Count**	**Pct**
Strongly Disagree	4	15.38%
Disagree	3	11.54%

Undecided	6	23.08%
**Agree**	**8**	**30.77%**
Strongly Agree	5	19.23%
**Aggregates**		
Count		26
Min		0
Max		4
Sum		59
Mean		2.27
Std Dev		1.34
Variance		1.8

Q40: You can get help in as little as 5 minutes here

Prompt	Count	Pct
Strongly Disagree	3	11.54%
Disagree	6	23.08%
Undecided	6	23.08%
**Agree**	**8**	**30.77%**
Strongly Agree	3	11.54%
**Aggregates**		
Count		26
Min		0
Max		4
Sum		54
Mean		2.08
Std Dev		1.23
Variance		1.51

Q41: You can get help in as little as 10 minutes here

Prompt	Count	Pct
Strongly Disagree	4	15.38%
Disagree	2	7.69%
Undecided	6	23.08%
**Agree**	**10**	**38.46%**
Strongly Agree	4	15.38%
**Aggregates**		
Count		26
Min		0
Max		4
Sum		60
Mean		2.31
Std Dev		1.29
Variance		1.66

Q42: That the developers of this program also uses these strategies being taught here

Prompt	Count	Pct
Strongly Disagree	3	11.54%
Disagree	5	19.23%
**Undecided**	**9**	**34.62%**
Agree	4	15.38%
Strongly Agree	5	19.23%
**Aggregates**		
Count		26

Min	0
Max	4
Sum	55
Mean	2.12
Std Dev	1.28
Variance	1.63

Q43: This program will be personalized to you		
**Prompt**	**Count**	**Pct**
Strongly Disagree	2	8.00%
Disagree	2	8.00%
Undecided	3	12.00%
**Agree**	**9**	**36.00%**
**Strongly Agree**	**9**	**36.00%**
**Aggregates**		
Count		25
Min		0
Max		4
Sum		71
Mean		2.84
Std Dev		1.25
Variance		1.56

Q44: This is a new and exciting intervention		
**Prompt**	**Count**	**Pct**
Strongly Disagree	6	23.08%
**Disagree**	**9**	**34.62%**
Undecided	3	11.54%
Agree	4	15.38%
Strongly Agree	4	15.38%
**Aggregates**		
Count		26
Min		0
Max		4
Sum		43
Mean		1.65
Std Dev		1.41
Variance		2

Q45: That there are videos of real people sharing their experiences here		
**Prompt**	**Count**	**Pct**
Strongly Disagree	3	11.54%
Disagree	5	19.23%
Undecided	3	11.54%
**Agree**	**9**	**34.62%**
Strongly Agree	6	23.08%
**Aggregates**		
Count		26
Min		0
Max		4
Sum		62
Mean		2.38

Std Dev	1.36
Variance	1.85

Q46: That becoming stronger as a result of life's difficulties is a focus here		
**Prompt**	**Count**	**Pct**
Strongly Disagree	4	15.38%
Disagree	4	15.38%
Undecided	4	15.38%
**Agree**	**7**	**26.92%**
**Strongly Agree**	**7**	**26.92%**
**Aggregates**		
Count		26
Min		0
Max		4
Sum		61
Mean		2.35
Std Dev		1.44
Variance		2.08

Q47: This information provided here looks slick and well made		
**Prompt**	**Count**	**Pct**
Strongly Disagree	7	26.92%
Disagree	7	26.92%
**Undecided**	**8**	**30.77%**
Agree	3	11.54%
Strongly Agree	1	3.85%
**Aggregates**		
Count		26
Min		0
Max		4
Sum		36
Mean		1.38
Std Dev		1.13
Variance		1.29

Q48: This intervention is professionally made		
**Prompt**	**Count**	**Pct**
**Strongly Disagree**	**6**	**23.08%**
Disagree	4	15.38%
**Undecided**	**6**	**23.08%**
Agree	4	15.38%
**Strongly Agree**	**6**	**23.08%**
**Aggregates**		
Count		26
Min		0
Max		4
Sum		52
Mean		2
Std Dev		1.5
Variance		2.24

CONTENTFREETEXT: What do you think would get people to visit an Online Intervention? You can make

up your own or copy one from above

Row	Comment
1	xyl
2	The know ledge that the intervention w ill not change any part of yourself or your life unless you allow it -- and it's completely private.
3	I w ould not bother w ith an impersonal online intervention. If the provider thinks there is a problem, I w ould prefer them to contact me personally.
4	That someone cares about you here
5	That this is intended to improve abilities to manage suicidal thoughts
6	Cheap and easily accessible support from doctors and other patients
7	The only w ay on online intervention w ould help me is if there w as a real person available to chat in real time. When I am feeling this aw ful, w atching videos online is not going to help. Getting an autogenerated message in my inbox w ould actually make me feel w orse because it implies that my provider doesn't care enough to contact me personally if they think there is a serious problem.
8	Your are not alone and you can feel better
9	That this is intended to improve abilities to manage suicidal thoughts
10	"That becoming stronger as a result of life's difficulties is a focus here"--focuses on a really positive goal.
11	That this is professionally made and organized, people care and that this is meant to provide one w ith tools
12	Three points, how ever w orded: There are others you can interact w ith struggling w ith the same issues; You're not alone; and w e care.
13	That you w ill be recognized as the best expert on yourself here
14	Others like you are looking for help too and w ill join you.
15	That there are videos of real people sharing their experiences here
16	Avoid using the term "intervention," maybe use something w ith a cheerier connotation. Like "pow -w ow ," "a group of your loved ones online," "a group of friends online," "support group online." The reason is because w hen I hear the term "intervention" is makes me think I'm going to be talked TO, not having an open dialogue. That's just my opinion though.
17	Chat support online
18	Nothing.
19	Let us help now .
20	Support developing life tools.
21	We care about you here and you are not alone
22	That there is a community of people in similar situations/w ith similar experiences as yourself and mental health professionals online w hich you can access at home at any time to find help, support and care
23	That there are videos of real people sharing their experiences here

VIDEO: General Thoughts about the Video

Row	Comment
1	It seems to be a video in the middle of a series. This doesn't feel personalized. It feels like a vague video addressing the surface issues of emotional instability w ith strange, unrelated space-age visual tricks intertw ined.
2	I couldn't concentrate on the message because the background w as kind of psychedelic/trippy. I didn't like the new agey, Sarah McLaclan-ish music.
3	did not get video on page (could not w atch, nothing w as there under "test video below "
4	Interesting special affects, w ith a morbid, sort of depressing tw ist. I w ould have liked to been able to hear the w oman speaking in the video. The music w as too loud to hear her. I'd rather the music be low er and the w oman's spiel louder. That w ould have been more interesting to me.
5	I thought the analogies and descriptions w ere easy to understand, and w hile I don't suffer from bipolar disorder, I identified w ith parts of the video
6	I didn't identify w ith the emotional problems described in the video. It makes a lot of assumptions regarding how the person if feeling. Not everyone w ho self injures or feels like life is not w orth living anymore is impulsive or fluctuates from one emotional extreme to the other. For me personally, I have been feeling bad for over half my life now and have tried over a dozen different meds w ith little success. I see no reason to expect that things w ill ever improve for me.
7	I had to w atch the beginning tw ice to totally understand to number scale. I w as not paying close enough attention the first time. I did enjoy the video overall. The imagery and music w ere quite nice. The video made me feel hopeful like there may be follow -up that w ill allow me to live in "the middle." It also made me feel not so alone. If someone is describing w hat I feel so clearly, other people must be feeling the same w ay. I didn't love the sauna/ice bath analogy. Analogies are very effective, but I believe a stronger one can be used. Good job over all.

8	The images w ere odd and not connected w ell to w hat w as being said. I also think the speaker needed to be identified w hen she first started talking. I have no idea if she is a researcher, mental health provider or actress. I think the script w as good.
9	unappealing visuals
10	I liked the aesthetic, how simply put the ideas w ere, and the music. It really calmed me dow n, actually. And I felt validated. I felt like my general existence w as at least superficially put up as simply different--rather than "inferior" or "broken."
11	Liked it. It w as a very candid and simplified w ay of understanding this particular state.
12	1) Liked the imagery and music w as okay. 2) "subjectively distressing" is w ording that comes across incredibly analytical rather than simple understanding, or empathy, or just descriptive of a place the 'consumer/patient' is at. 3) Metaphor of sauna and cold tub seems over used.
13	Watching this video w as a very positive experience.
14	it makes very valid points about tw o polar extremes, but I feel like the animation is too dark for one thing (hard to see, so is the lady speaking) and I felt like it didn't draw me into w hat w as being said, seemed to be a distraction.
15	I like the w ay this w as done. I like that the w oman w asn't show n the w hole time. Using music, and and voice at the same time w as good, but I think the music w as a little too loud, it w as a distraction from the spoken w ords. The sound w ould stop then go. Sort of like a hiccup
16	I like the numbers, it makes it feel more concrete. Good and relaxing background music. There w ere more than one thing that w as said that made me think "that's how I feel."
17	Getting therapy is good.
18	I liked the visuals. Had to w atch tw ice to get all the information. The background music felt appropriate.
19	THought the message w ent w ell w ith the abstract graphics and seemed to convey an understading of the problems associated w ith depression.
20	The music w as too loud and interfered w ith my ability to hear all being said. the question being asked w as hard to hear and the text w as not up long enough to register. for the most part the graphics w ere interesting, but I tended to look at them rather than listening to w hat w as being said.
21	I liked the music, the film w as very artistic and beautifully made. It w as hard for me to focus on w hat the w oman w as saying. I found the music and the imagery to be a bit distracting and it w as taking up most of my attention. I really enjoyed this video overall, but, perhaps it w ould be more useful to those patients w ith ADHD or attention problems (like myself) if there w asn't quite as much going on in the background. That being said, I thought that the overall atmosphere of the video w as very calm, peaceful and relaxing. I feel like it w as easy to w atch (as compared w ith some of the other, more "boring" videos I've seen explaining mental health and emotions) and very w ell made, brilliantly artistic. I liked the tranquil quality it had.
22	The video didn't w ork

Q49: I could relate to the video

Prompt	Count	Pct
Strongly Disagree	0	.00%
Disagree	1	4.35%
Undecided	7	30.43%
**Agree**	**8**	**34.78%**
Strongly Agree	7	30.43%
Video Didn't Work	0	.00%
**Aggregates**		
Count		23
Min		1
Max		4
Sum		67
Mean		2.91
Std Dev		0.9
Variance		0.81

Q50: I understood the video on the first watch

Prompt	Count	Pct
Strongly Disagree	1	4.35%
**Disagree**	**7**	**30.43%**
Undecided	3	13.04%
**Agree**	**7**	**30.43%**
Strongly Agree	5	21.74%

Video Didn't Work	0	.00%
**Aggregates**		
Count		23
Min		0
Max		4
Sum		54
Mean		2.35
Std Dev		1.27
Variance		1.6

Q51: The sound on the video was clear		
**Prompt**	**Count**	**Pct**
Strongly Disagree	1	4.35%
Disagree	2	8.70%
Undecided	2	8.70%
**Agree**	**12**	**52.17%**
Strongly Agree	5	21.74%
Video Didn't Work	1	4.35%
**Aggregates**		
Count		23
Min		-99
Max		4
Sum		-37
Mean		-1.61
Std Dev		21.26
Variance		451.79

Q52: I wanted to watch the video again because I was interested		
**Prompt**	**Count**	**Pct**
Strongly Disagree	1	4.35%
**Disagree**	**6**	**26.09%**
Undecided	5	21.74%
**Agree**	**6**	**26.09%**
Strongly Agree	4	17.39%
Video Didn't Work	1	4.35%
**Aggregates**		
Count		23
Min		-99
Max		4
Sum		-49
Mean		-2.13
Std Dev		21.15
Variance		447.3

Q53: I liked the visuals		
**Prompt**	**Count**	**Pct**
Strongly Disagree	2	8.70%
Disagree	2	8.70%
Undecided	5	21.74%
Agree	6	26.09%
**Strongly Agree**	**7**	**30.43%**
Video Didn't Work	1	4.35%
**Aggregates**		

Count	23
Min	-99
Max	4
Sum	-41
Mean	-1.78
Std Dev	21.23
Variance	450.72

Q54: I liked the music

Prompt	Count	Pct
Strongly Disagree	1	4.35%
Disagree	2	8.70%
Undecided	5	21.74%
Agree	7	30.43%
**Strongly Agree**	**8**	**34.78%**
Video Didn't Work	0	.00%
**Aggregates**		
Count		23
Min		0
Max		4
Sum		65
Mean		2.83
Std Dev		1.15
Variance		1.33

Q55: I liked what the speaker had to say

Prompt	Count	Pct
Strongly Disagree	0	.00%
Disagree	1	4.35%
**Undecided**	**9**	**39.13%**
Agree	5	21.74%
Strongly Agree	8	34.78%
Video Didn't Work	0	.00%
**Aggregates**		
Count		23
Min		1
Max		4
Sum		66
Mean		2.87
Std Dev		0.97
Variance		0.94

Q56: I feel validated about my experience

Prompt	Count	Pct
Strongly Disagree	0	.00%
Disagree	3	13.04%
**Undecided**	**11**	**47.83%**
Agree	5	21.74%
Strongly Agree	4	17.39%
Video Didn't Work	0	.00%
**Aggregates**		
Count		23

Min	1
Max	4
Sum	56
Mean	2.43
Std Dev	0.95
Variance	0.89

Q26: Now Matters Now

Prompt	Count	Pct
Strongly Disagree	2	9.09%
Disagree	2	9.09%
**Undecided**	**8**	**36.36%**
Agree	5	22.73%
Strongly Agree	5	22.73%
**Aggregates**		
Count		22
Min		0
Max		4
Sum		53
Mean		2.41
Std Dev		1.22
Variance		1.49

Q57: The Next Steps

Prompt	Count	Pct
Strongly Disagree	2	8.70%
Disagree	3	13.04%
**Undecided**	**8**	**34.78%**
**Agree**	**8**	**34.78%**
Strongly Agree	2	8.70%
**Aggregates**		
Count		23
Min		0
Max		4
Sum		51
Mean		2.22
Std Dev		1.09
Variance		1.18

Q58: Wayfind

Prompt	Count	Pct
Strongly Disagree	5	22.73%
**Disagree**	**7**	**31.82%**
Undecided	5	22.73%
Agree	5	22.73%
Strongly Agree	0	.00%
**Aggregates**		
Count		22
Min		0
Max		3
Sum		32
Mean		1.45
Std Dev		1.1

Variance		1.21

Q59: Beyond Basics		
**Prompt**	**Count**	**Pct**
Strongly Disagree	4	17.39%
**Disagree**	**7**	**30.43%**
Undecided	3	13.04%
Agree	5	21.74%
Strongly Agree	4	17.39%
**Aggregates**		
Count		23
Min		0
Max		4
Sum		44
Mean		1.91
Std Dev		1.41
Variance		1.99

Q60: What Defines You		
**Prompt**	**Count**	**Pct**
Strongly Disagree	2	9.09%
**Disagree**	**7**	**31.82%**
**Undecided**	**7**	**31.82%**
Agree	4	18.18%
Strongly Agree	2	9.09%
**Aggregates**		
Count		22
Min		0
Max		4
Sum		41
Mean		1.86
Std Dev		1.13
Variance		1.27

Q61: You Are Here		
**Prompt**	**Count**	**Pct**
Strongly Disagree	0	.00%
Disagree	6	26.09%
Undecided	4	17.39%
**Agree**	**10**	**43.48%**
Strongly Agree	3	13.04%
**Aggregates**		
Count		23
Min		1
Max		4
Sum		56
Mean		2.43
Std Dev		1.04
Variance		1.08

Q62: Tiny Matters		
**Prompt**	**Count**	**Pct**

Strongly Disagree	6	26.09%
Disagree	6	26.09%
**Undecided**	**7**	**30.43%**
Agree	3	13.04%
Strongly Agree	1	4.35%
**Aggregates**		
Count		23
Min		0
Max		4
Sum		33
Mean		1.43
Std Dev		1.16
Variance		1.35

Q63: Make Your Moment

Prompt	Count	Pct
Strongly Disagree	2	8.70%
Disagree	4	17.39%
Undecided	6	26.09%
**Agree**	**9**	**39.13%**
Strongly Agree	2	8.70%
**Aggregates**		
Count		23
Min		0
Max		4
Sum		51
Mean		2.22
Std Dev		1.13
Variance		1.27

Q64: Here You Are

Prompt	Count	Pct
Strongly Disagree	3	13.04%
**Disagree**	**7**	**30.43%**
Undecided	4	17.39%
**Agree**	**7**	**30.43%**
Strongly Agree	2	8.70%
**Aggregates**		
Count		23
Min		0
Max		4
Sum		44
Mean		1.91
Std Dev		1.24
Variance		1.54

LOGO_STYLE: Which do you prefer of the above logo styles?

Prompt	Count	Pct
**Rectangle Version**	**12**	**54.55%**
Circle Version	10	45.45%
**Aggregates**		
Count		22
Min		0

Max	1
Sum	10
Mean	0.45
Std Dev	0.51
Variance	0.26

DATSTAT.SUBMISSIONID: Unique id of this data submission.		
**Prompt**	**Count**	**Pct**
**Aggregates**		
Count		56
Min		1
Max		57
Sum		1611
Mean		28.77
Std Dev		16.65
Variance		277.38

DATSTAT.SESSIONID: Session Id.		
**Prompt**	**Count**	**Pct**
**Aggregates**		
Count		56

DATSTAT.VERSION: The version of the survey used.		
**Prompt**	**Count**	**Pct**
**Aggregates**		
Count		56
Min		1
Max		3
Sum		151
Mean		2.7
Std Dev		0.71
Variance		0.51

DATSTAT.LOCALE: The preferred locale of the participant/user.		
**Prompt**	**Count**	**Pct**
**Aggregates**		
Count		56
Min		1033
Max		1033
Sum		57848
Mean		1033
Std Dev		0
Variance		0

DATSTAT.LANGUAGE: The survey language used by this participant.		
**Prompt**	**Count**	**Pct**
**en-US**	**56**	**100.00%**
**Aggregates**		
Count		56

DATSTAT.BROWSER: The browser this participant/user used to enter data.

Row	Comment
1	Mozilla/5.0 (Windows NT 5.1) AppleWebKit/537.17 (KHTML, like Gecko) Chrome/24.0.1312.57 Safari/537.17
2	Mozilla/5.0 (Linux; U; Android 4.1.1; en-us; EVO Build/JRO03C) AppleWebKit/534.30 (KHTML, like Gecko) Version/4.0 Mobile Safari/534.30
3	Mozilla/5.0 (iPhone; CPU iPhone OS 6_0 like Mac OS X) AppleWebKit/536.26 (KHTML, like Gecko) Mobile/10A403
4	Mozilla/5.0 (Windows NT 5.1; rv:18.0) Gecko/20100101 Firefox/18.0
5	Mozilla/5.0 (iPhone; CPU iPhone OS 6_0 like Mac OS X) AppleWebKit/536.26 (KHTML, like Gecko) Mobile/10A403
6	Mozilla/5.0 (iPhone; CPU iPhone OS 6_0 like Mac OS X) AppleWebKit/536.26 (KHTML, like Gecko) Mobile/10A403
7	Mozilla/5.0 (iPhone; CPU iPhone OS 6_0 like Mac OS X) AppleWebKit/536.26 (KHTML, like Gecko) Mobile/10A403
8	Mozilla/5.0 (iPhone; CPU iPhone OS 6_0 like Mac OS X) AppleWebKit/536.26 (KHTML, like Gecko) Mobile/10A403
9	Mozilla/5.0 (Windows NT 5.1) AppleWebKit/537.22 (KHTML, like Gecko) Chrome/25.0.1364.152 Safari/537.22
10	Mozilla/5.0 (Linux; U; Android 4.1.1; en-us; EVO Build/JRO03C) AppleWebKit/534.30 (KHTML, like Gecko) Version/4.0 Mobile Safari/534.30
11	Mozilla/4.0 (compatible; MSIE 7.0; Windows NT 5.1; Trident/4.0; .NET CLR 1.1.4322; .NET CLR 1.0.3705; .NET CLR 2.0.50727; .NET CLR 3.0.4506.2152; .NET CLR 3.5.30729; .NET4.0C; .NET4.0E; MS-RTC LM 8)
12	Mozilla/5.0 (iPhone; CPU iPhone OS 6_1_1 like Mac OS X) AppleWebKit/536.26 (KHTML, like Gecko) Mobile/10B145 iPhone4,1 BingWeb/3.03.1428.20120423
13	Mozilla/5.0 (iPhone; CPU iPhone OS 6_1_1 like Mac OS X) AppleWebKit/536.26 (KHTML, like Gecko) Mobile/10B145 iPhone4,1 BingWeb/3.03.1428.20120423
14	Mozilla/5.0 (Windows NT 5.1) AppleWebKit/537.22 (KHTML, like Gecko) Chrome/25.0.1364.152 Safari/537.22
15	Mozilla/4.0 (compatible; MSIE 7.0; Windows NT 6.1; WOW64; Trident/4.0; .NET CLR 1.1.4322; .NET CLR 2.0.50727; .NET CLR 3.0.30729; .NET CLR 3.5.30729; .NET4.0C; .NET4.0E; MS-RTC LM 8; SLCC2)
16	Mozilla/4.0 (compatible; MSIE 7.0; Windows NT 5.1; Trident/4.0; .NET CLR 1.1.4322; .NET CLR 1.0.3705; .NET CLR 2.0.50727; .NET CLR 3.0.4506.2152; .NET CLR 3.5.30729; .NET4.0C; .NET4.0E; MS-RTC LM 8)
17	Mozilla/5.0 (Windows NT 6.1; WOW64) AppleWebKit/537.22 (KHTML, like Gecko) Chrome/25.0.1364.152 Safari/537.22
18	Mozilla/5.0 (iPhone; CPU iPhone OS 6_1_2 like Mac OS X) AppleWebKit/536.26 (KHTML, like Gecko) Mobile/10B146
19	Mozilla/5.0 (iPhone; CPU iPhone OS 6_1_2 like Mac OS X) AppleWebKit/536.26 (KHTML, like Gecko) Mobile/10B146
20	Mozilla/5.0 (iPad; CPU OS 6_1_2 like Mac OS X) AppleWebKit/536.26 (KHTML, like Gecko) Version/6.0 Mobile/10B146 Safari/8536.25
21	Mozilla/5.0 (iPhone; CPU iPhone OS 6_0_1 like Mac OS X) AppleWebKit/536.26 (KHTML, like Gecko) Version/6.0 Mobile/10A525 Safari/8536.25
22	Mozilla/5.0 (iPhone; CPU iPhone OS 6_0 like Mac OS X) AppleWebKit/536.26 (KHTML, like Gecko) Mobile/10A403
23	Mozilla/5.0 (compatible; MSIE 10.0; Windows NT 6.2; WOW64; Trident/6.0; Touch; MALCJS)
24	Mozilla/5.0 (Windows NT 5.1; rv:19.0) Gecko/20100101 Firefox/19.0
25	Mozilla/5.0 (Windows NT 6.1) AppleWebKit/537.22 (KHTML, like Gecko) Chrome/25.0.1364.172 Safari/537.22
26	Mozilla/5.0 (iPad; CPU OS 6_1_2 like Mac OS X) AppleWebKit/536.26 (KHTML, like Gecko) Version/6.0 Mobile/10B146 Safari/8536.25
27	Mozilla/5.0 (Macintosh; Intel Mac OS X 10.5; rv:12.0) Gecko/20100101 Firefox/12.0
28	Mozilla/5.0 (Linux; U; Android 4.1.1; en-us; DROID RAZR HD Build/9.8.1Q_39) AppleWebKit/534.30 (KHTML, like Gecko) Version/4.0 Mobile Safari/534.30
29	Mozilla/5.0 (Macintosh; Intel Mac OS X 10_8_2) AppleWebKit/536.26.17 (KHTML, like Gecko) Version/6.0.2 Safari/536.26.17
30	Mozilla/5.0 (Linux; U; Android 4.0.4; en-us; C5155 Build/IML77) AppleWebKit/534.30 (KHTML, like Gecko) Version/4.0 Mobile Safari/534.30
31	Mozilla/5.0 (compatible; MSIE 9.0; Windows NT 6.1; WOW64; Trident/5.0; FunWebProducts)
32	Mozilla/5.0 (Macintosh; Intel Mac OS X 10_7_5) AppleWebKit/536.28.10 (KHTML, like Gecko) Version/6.0.3 Safari/536.28.10
33	Mozilla/5.0 (Windows NT 6.1) AppleWebKit/537.22 (KHTML, like Gecko) Chrome/25.0.1364.172 Safari/537.22
34	Mozilla/5.0 (iPhone; CPU iPhone OS 6_1_2 like Mac OS X) AppleWebKit/536.26 (KHTML, like Gecko) Version/6.0 Mobile/10B146 Safari/8536.25
35	Mozilla/4.0 (compatible; MSIE 7.0; Windows NT 6.1; WOW64; Trident/4.0; .NET CLR 1.1.4322; .NET CLR 2.0.50727; .NET CLR 3.0.30729; .NET CLR 3.5.30729; .NET4.0C; .NET4.0E; MS-RTC LM 8; SLCC2)
36	Mozilla/5.0 (Windows NT 5.1) AppleWebKit/537.22 (KHTML, like Gecko) Chrome/25.0.1364.172 Safari/537.22
37	Mozilla/5.0 (Windows NT 5.1) AppleWebKit/537.22 (KHTML, like Gecko) Chrome/25.0.1364.172 Safari/537.22
38	Mozilla/5.0 (iPhone; CPU iPhone OS 6_1_2 like Mac OS X) AppleWebKit/536.26 (KHTML, like Gecko) Mobile/10B146
39	Mozilla/5.0 (iPhone; CPU iPhone OS 6_0_1 like Mac OS X) AppleWebKit/536.26 (KHTML, like Gecko) Mobile/10A523
40	Mozilla/5.0 (Macintosh; U; Intel Mac OS X 10.6; en-US; rv:1.9.2.13) Gecko/20101203 Firefox/3.6.13
41	Mozilla/5.0 (Windows NT 6.1; WOW64) AppleWebKit/537.22 (KHTML, like Gecko) Chrome/25.0.1364.172 Safari/537.22

42	Mozilla/5.0 (Window s NT 5.1) AppleWebKit/537.22 (KHTML, like Gecko) Chrome/25.0.1364.172 Safari/537.22
43	Mozilla/5.0 (iPhone; CPU iPhone OS 6_1_3 like Mac OS X) AppleWebKit/536.26 (KHTML, like Gecko) Mobile/10B329
44	Mozilla/5.0 (iPhone; CPU iPhone OS 6_1_3 like Mac OS X) AppleWebKit/536.26 (KHTML, like Gecko) Mobile/10B329
45	Mozilla/5.0 (iPhone; CPU iPhone OS 6_1_3 like Mac OS X) AppleWebKit/536.26 (KHTML, like Gecko) Mobile/10B329
46	Mozilla/5.0 (iPad; CPU OS 6_1_3 like Mac OS X) AppleWebKit/536.26 (KHTML, like Gecko) Mobile/10B329
47	Mozilla/5.0 (iPhone; CPU iPhone OS 5_1_1 like Mac OS X) AppleWebKit/534.46 (KHTML, like Gecko) Mobile/9B206
48	Mozilla/5.0 (Window s NT 6.1; WOW64; rv:19.0) Gecko/20100101 Firefox/19.0
49	Mozilla/5.0 (iPad; U; CPU OS 4_3_3 like Mac OS X; en-us) AppleWebKit/533.17.9 (KHTML, like Gecko) Version/5.0.2 Mobile/8J3 Safari/6533.18.5
50	Mozilla/5.0 (Linux; U; Android 4.0.4; en-us; SPH-D710BST Build/IMM76I) AppleWebKit/534.30 (KHTML, like Gecko) Version/4.0 Mobile Safari/534.30
51	Mozilla/5.0 (Linux; U; Android 4.0.4; en-us; C5170 Build/IML77) AppleWebKit/534.30 (KHTML, like Gecko) Version/4.0 Mobile Safari/534.30
52	Mozilla/5.0 (Linux; U; Android 4.0.4; en-us; C5170 Build/IML77) AppleWebKit/534.30 (KHTML, like Gecko) Version/4.0 Mobile Safari/534.30
53	Mozilla/5.0 (Window s NT 6.1) AppleWebKit/537.31 (KHTML, like Gecko) Chrome/26.0.1410.43 Safari/537.31
54	Mozilla/5.0 (Macintosh; Intel Mac OS X 10.7; rv:19.0) Gecko/20100101 Firefox/19.0
55	Mozilla/5.0 (iPhone; CPU iPhone OS 6_1_3 like Mac OS X) AppleWebKit/536.26 (KHTML, like Gecko) Version/6.0 Mobile/10B329 Safari/8536.25
56	Mozilla/4.0 (compatible; MSIE 8.0; Window s NT 5.1; Trident/4.0; GTB7.4; .NET CLR 1.1.4322; .NET CLR 2.0.50727; .NET CLR 3.0.4506.2152; .NET CLR 3.5.30729; .NET4.0C; .NET4.0E; AskTbORJ/5.15.15.36191)

DATSTAT.STARTDATETIME: The date and time this participant/user began entering data.

Prompt	Count	Pct
**Aggregates**		
Count		56
Min		2/22/2013 12:43:00 PM
Max		4/12/2013 5:00:32 PM

DATSTAT.ENDDATETIME: The date and time this participant/user finished entering data.

Prompt	Count	Pct
**Aggregates**		
Count		56
Min		2/22/2013 12:43:00 PM
Max		4/12/2013 5:14:38 PM

DATSTAT.ELAPSEDTIME: The total number of minutes it took this participant/user to finish entering data.

Prompt	Count	Pct
**Aggregates**		
Count		56
Min		1
Max		138
Sum		728
Mean		13
Std Dev		22.04
Variance		485.56

DATSTAT.SUBMISSIONSTATUS: Describes how the data set was submitted.

Prompt	Count	Pct
Completed	25	44.64%
**Partial**	**31**	**55.36%**
Not started	0	.00%
Terminated	0	.00%

**Aggregates**	
Count	56
Min	1
Max	2
Sum	87
Mean	1.55
Std Dev	0.5
Variance	0.25

DATSTAT.LOGINCOUNT: Number of times the participant has logged in.

Prompt	Count	Pct
**Aggregates**		
Count		56
Min		1
Max		1
Sum		56
Mean		1
Std Dev		0
Variance		0

DATSTAT.JAVASCRIPT: JavaScript enabled?

Prompt	Count	Pct
No	0	.00%
**Yes**	**56**	**100.00%**
**Aggregates**		
Count		56

DATSTAT.TIMEPERIOD: The time period of this submission.

Prompt	Count	Pct
**Version 1**	**56**	**100.00%**
**Aggregates**		
Count		56
Min		1
Max		1
Sum		56
Mean		1
Std Dev		0
Variance		0

DATSTAT.PCTCOMPLETE: Percent complete.

Prompt	Count	Pct
**Aggregates**		
Count		56
Min		0
Max		100
Sum		3002
Mean		53.61
Std Dev		47.74
Variance		2279.26

DATSTAT.LOGINDATETIME: The date and time when this participant last logged in.

Prompt	Count	Pct

Aggregates	
Count	56
Min	2/22/2013 12:43:00 PM
Max	4/12/2013 5:00:32 PM

DATSTAT.UPLOADDATETIME: The date and time if and when this submission was uploaded.

Prompt	Count	Pct
**Aggregates**		
Count		0

DATSTAT.UPLOADUSER: The name of the user who uploaded this submission.

Row	Comment

DATSTAT.UPLOADTYPE: The type of upload.

Prompt	Count	Pct
**Remote data collection**	**0**	**.00%**
**Imported**	**0**	**.00%**
**Aggregates**		
Count		0
Sum		0
Mean		0
Std Dev		0
Variance		0

DATSTAT.NUMPRESENTED: Number of questions presented to the participant.

Prompt	Count	Pct
**Aggregates**		
Count		56
Min		1
Max		75
Sum		2794
Mean		49.89
Std Dev		35.35
Variance		1249.88

DATSTAT.NUMANSWERED: Number of questions answered by this participant.

Prompt	Count	Pct
**Aggregates**		
Count		56
Min		0
Max		75
Sum		1958
Mean		34.96
Std Dev		35.68
Variance		1273.31

DATSTAT.NUMUNANSWERED: Number of questions unanswered by this participant.

Prompt	Count	Pct
**Aggregates**		
Count		56
Min		0

Max	74
Sum	836
Mean	14.93
Std Dev	27.17
Variance	738.25

DATSTAT.PCTUNANSWERED: Percentage of questions unanswered by this participant.		
**Prompt**	**Count**	**Pct**
**Aggregates**		
Count		56
Min		0
Max		100
Sum		2881
Mean		51.45
Std Dev		47.65
Variance		2270.51

DATSTAT.SITE: Participant site at the time when this survey was started.		
**Prompt**	**Count**	**Pct**
**Aggregates**		
Count		0

DATSTAT.INTERVIEWER: User that interviewed the participant.		
**Prompt**	**Count**	**Pct**
**Aggregates**		
Count		0
